# Editor's note: new biomaterials for dental and craniomaxillofacial applications section

**DOI:** 10.1186/s13005-015-0093-6

**Published:** 2015-11-04

**Authors:** Thomas Stamm

**Affiliations:** University of Münster, Münster, Germany

**Fig. 1:**
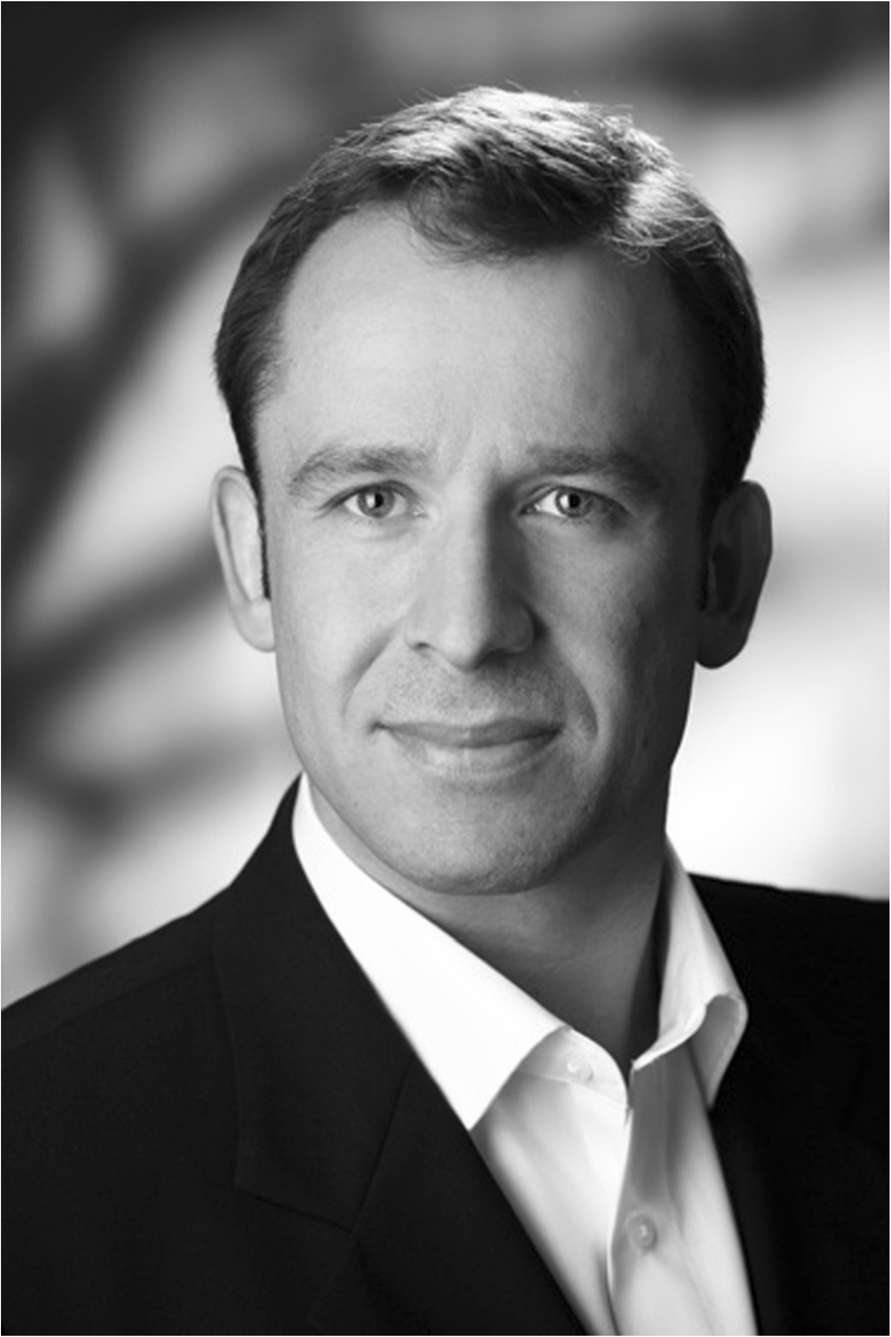
Dr. Jörg Neunzehn, scientist and group leader at the Chair for Biomaterials of the Technische Universität Dresden, Germany

The Journal Development Committee of *Head & Face Medicine* has decided to create the new section “Biomaterials for dental and craniomaxillofacial applications”. As editors of *Head & Face Medicine* we are glad that Dr. Jörg Neunzehn, scientist and group leader at the Chair for Biomaterials of the Technische Universität Dresden, accepted this new editorial board position and that he will offer his outstanding expertise to work with us (Fig. 1).

Jörg Neunzehn is lecturer and researcher on the field of dental materials and biomaterials, especially for bone graft materials, dental tissue-engineering and nano-analytics.

Based on his education as dental technician, his materials scientific study of dental technologies, his scientific experiences during his work at the department of craniomaxillofacial surgery at the Westfälische-Wilhelms-Universität Münster and his current position as group leader for “dental materials and nano-analytic” of the Chair for Biomaterials at the Technische Universität Dresden he is a qualified new member for our Editorial Board.

His actual research interests focus on the development of bioinspired bone graft materials, materials for dental, oral and facial tissue regeneration and tissue engineering, new materials for dental applications and surface analytics.

*Head & Face Medicine* is grateful that Dr. Neunzehn will spend his valuable time to hold this new Section Editor position. His contribution will be important in helping shape the journal's course over the coming years.

